# Changes in surface hydrology, soil moisture and gross primary production in the Amazon during the 2015/2016 El Niño

**DOI:** 10.1098/rstb.2018.0084

**Published:** 2018-10-08

**Authors:** Erik van Schaik, Lars Killaars, Naomi E. Smith, Gerbrand Koren, L. P. H. van Beek, Wouter Peters, Ingrid T. van der Laan-Luijkx

**Affiliations:** 1 Department of Meteorology and Air Quality, Wageningen University and Research, Wageningen, The Netherlands; 2 Centre for Isotope Research, University of Groningen, Centre for Isotope Research, Groningen, The Netherlands; 3 Department of Physical Geography, Faculty of Geosciences, Utrecht University, Utrecht, The Netherlands

**Keywords:** El Niño, tropical terrestrial carbon cycle, river discharge, soil moisture, gross primary productivity, Amazon

## Abstract

The 2015/2016 El Niño event caused severe changes in precipitation across the tropics. This impacted surface hydrology, such as river run-off and soil moisture availability, thereby triggering reductions in gross primary production (GPP). Many biosphere models lack the detailed hydrological component required to accurately quantify anomalies in surface hydrology and GPP during droughts in tropical regions. Here, we take the novel approach of coupling the biosphere model SiBCASA with the advanced hydrological model PCR-GLOBWB to attempt such a quantification across the Amazon basin during the drought in 2015/2016. We calculate 30–40% reduced river discharge in the Amazon starting in October 2015, lagging behind the precipitation anomaly by approximately one month and in good agreement with river gauge observations. Soil moisture shows distinctly asymmetrical spatial anomalies with large reductions across the north-eastern part of the basin, which persisted into the following dry season. This added to drought stress in vegetation, already present owing to vapour pressure deficits at the leaf, resulting in a loss of GPP of 0.95 (0.69 to 1.20) PgC between October 2015 and March 2016 compared with the 2007–2014 average. Only 11% (10–12%) of the reduction in GPP was found in the (wetter) north-western part of the basin, whereas the north-eastern and southern regions were affected more strongly, with 56% (54–56%) and 33% (31–33%) of the total, respectively. Uncertainty on this anomaly mostly reflects the unknown rooting depths of vegetation.

This article is part of a discussion meeting issue ‘The impact of the 2015/2016 El Niño on the terrestrial tropical carbon cycle: patterns, mechanisms and implications’.

## 1. Introduction

The tropical latitudes are covered by a large amount of the world's vegetation and have high carbon stocks both above- and below-ground [1]. These regions therefore play an important role in the global carbon budget [2,3]. The carbon uptake by tropical forests shows large interannual variability [4] and is one of the main sources of uncertainty in climate models [5,6]. One driver of this variability is the occurrence of extended drought periods, during which low rainfall leads to a decrease in soil moisture levels [7]. This limit on the water available to vegetation can reduce transpiration and photosynthesis, further reducing the water available for precipitation by atmospheric recycling [8–10].

The Amazon region has experienced severe droughts in recent years, including in 2005 [11], 2010 [12] and the recent 2015/2016 El Niño period, which had significant effects across the tropics, including the Amazon basin [13–15]. Liu *et al.* [16] found that the annual mean precipitation during the 2015/2016 drought was the lowest in 35 years and that the annual mean precipitation was 3 s.d. lower in 2015 relative to 2011. Yang *et al.* [17] noted a significant decrease in river discharge and signs of a hydrological drought in terrestrial water storage during this latest El Niño event. Jiménez-Muñoz *et al.* [15] suggested that the drought was limited to the eastern Amazon basin after analysing ERA-Interim precipitation observations, whereas Yang *et al.* [17] found decreased precipitation across a much broader area from two alternative precipitation datasets together with observations of river discharge and terrestrial water storage. Both studies confirm that the Amazon region experienced an intense drought during the 2015/2016 El Niño period.

The response of the vegetation in the Amazon to droughts leads to reductions in carbon uptake by the biosphere [11,17–20] and an increase in emissions from fires [21–23]. During the 2010 drought, there was a significant reduction in net ecosystem production (NEP) over the Amazon basin of 0.08 to 0.28 PgC yr^−1^ compared with 2011 [18,24,25], which together with increased fires (0.16 to 0.43 PgC yr^−1^) strongly increased carbon release to the atmosphere. Liu *et al.* [16] assessed the drought impact in 2015/2016 and found that in the drought-affected parts of the Amazon, NEP decreased by 0.9 ± 0.24 PgC during 2015 compared with 2011. As was also seen during the 2005 drought [26], the availability of more sunlight during the drought led to an increase in ‘greenness’ during 2015 [17]. At the same time, sun-induced fluorescence (SIF), a measure for photosynthetic activity, was significantly decreased across the basin [17,27], indicating that photosynthesis can be decoupled from canopy greenness. These studies demonstrate that the response of tropical ecosystems to droughts is not well understood and also varies between regions [28].

In this paper, we aim to quantify the impact of the 2015/2016 El Niño period on the carbon uptake in the Amazon. We aim to calculate the impact of reduced precipitation on surface hydrology and soil moisture and subsequently on the photosynthetic carbon uptake, the gross primary production (GPP), across the full basin at high resolution. To estimate the carbon exchange of the Amazon, we use the terrestrial biosphere model SiBCASA, which is a combination of the Simple Biosphere (SiB) model and the biogeochemistry of the Carnegie–Ames–Stanford Approach (CASA) [29,30]. We couple SiBCASA to the hydrological model PCRaster GLOBal Water Balance (PCR-GLOBWB) [31] to account for one of the main limitations of the SiBCASA model, which is the too low response to soil moisture stress [32,33]. This is a known uncertainty in terrestrial biosphere models in general and leads to large differences in their estimated carbon cycle drought response [32,34]. Van der Laan-Luijkx *et al.* [24] showed for the Amazon region that the default SiBCASA model did not see any effect on the net carbon uptake during the major Amazon drought in 2010. In this paper, we propose a new method to improve on this limitation by direct coupling with the surface hydrology and soil moisture balance from our hydrological model. The use of these models allows us to specifically assess the soil moisture stress placed on the Amazon vegetation during the drought.

We first describe the SiBCASA and PCR-GLOBWB models in §2. Subsequently, we describe the results on the hydrological balance and the carbon balance of the Amazon in §3, followed by a discussion of the results and our conclusion.

## 2. Material and methods

### (a) PCRaster GLOBal Water Balance

The global hydrological model PCR-GLOBWB 2 [31] simulates the hydrology globally with a spatial resolution of 5 arcmin and a daily time step. In this paper, we focus on the Amazon basin specifically, while our simulations extend across most of the South American continent and are used for validation (see electronic supplementary material). The model contains two soil layers: an underlying groundwater layer, and snow and vegetation canopy layers. Vertical interaction is possible between these layers, but there is no direct horizontal exchange of water between the different cells; excess surface or soil water is routed along a river network using the kinematic wave method with a time step of approximately 20 min. PCR-GLOBWB is parameterized on the basis of existing global datasets and is not further calibrated to a specific meteorological input product, to maintain the option to independently assess various products.

#### (i) Meteorological forcing data

PCR-GLOBWB uses daily time series of precipitation, temperature and reference evaporation as meteorological drivers of the model. Precipitation determines the input of water in the hydrological system and is therefore one of the most important drivers of the model and an important source of uncertainty in hydrological modelling [35–37]. We have therefore used three alternative precipitation datasets in our simulations. These are: MSWEP (Multi-Source Weighted-Ensemble Precipitation v. 2.1, [38]), ERA5 (from the European Centre for Medium-range Weather Forecasts (ECMWF) [39]) and Tropical Rainfall Measuring Mission (TRMM) Multi-satellite Precipitation Analysis (TMPA) 3B42 v. 7 [40]. We use these three simulations to determine the uncertainty range following from the choice of precipitation input data. For the subsequent analysis, we have selected the simulations with the MSWEP precipitation input data, as they show the best comparison with independent discharge observations between the simulations with the three precipitation datasets (see §3). MSWEP also has the longest time record, and includes actual precipitation observations, which is not the case for TRMM or ERA5 [39,40].

We have used temperature and reference evaporation data based on monthly data from the Climate Research Unit (CRU) TS dataset downscaled to daily values [41]. We have used v. 3.2 for the period before 2010 and v. 4.01 for 2010–2016. The downscaling procedure for the data before 2010 is described in Sutanudjaja *et al.* [31]. In the period after 2010, the daily variance of the ERA5 temperature and reference evaporation is added to the CRU TS monthly means.

#### (ii) Validation

**Figure 1.**
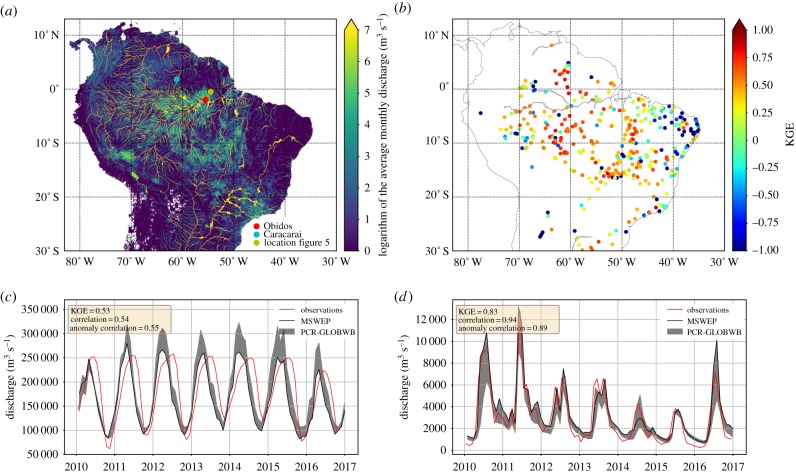
(*a*) Mean monthly discharge over the Amazon basin as simulated with PCR-GLOBWB with MSWEP precipitation. (*b*) KGE (see Material and methods for explanation) model skill scores of the PCR-GLOBWB–MSWEP simulation for discharge stations across the Amazon basin (+1 indicates the highest skill score); observed and simulated discharge time series at (*c*) Obidos and (*d*) Caracarai. The locations of these stations are shown in (*a*). The grey bands in (*c*) and (*d*) indicate the minimum and maximum of the three different PCR-GLOBWB simulations with MSWEP, TRMM and ERA5 precipitation driver data, respectively. The black line indicates the simulation using MSWEP data.

We compared the monthly results of PCR-GLOBWB with discharge measurements from the Global Runoff Data Centre [42] and the HYBAM dataset (www.ore-hybam.org). We extended the validation of PCR-GLOBWB presented in Sutanudjaja *et al.* [31] by validating our results with observations from 360 stations across the Amazon for recent years (see figure 1*b* for their locations). We have calculated the Kling–Gupta Efficiency (KGE) score for each station, which is a standard measure of performance in hydrological modelling and equally measures timing and amplitude differences and model bias. It returns a single score from –∞ to 1, where 1 is a perfect match and negative values indicate poor model performance [43,44]. Further details of the model set-up and results of the validation can be found in the electronic supplementary material. We also compare our results with the terrestrial water storage from GRACE [45] from the JPL-RL05M mascon product by the Jet Propulsion Laboratory (JPL).

### (b) SiBCASA

The biosphere model SiBCASA combines the photosynthesis parameterizations of the SiB [46] model with the biogeochemistry of the Carnegie–Ames–Stanford Approach (CASA) [47]. SiBCASA has been separately described and validated with flux measurements [29], extended with ^13^C isotope kinetics and compared with other biosphere models [30] and used to investigate soil moisture limitations [33] as well as changes in water-use efficiency [32]. It explicitly differentiates between C3 and C4 vegetation following the enzyme kinetics of Farquhar *et al.* [48] (C3) and Collatz *et al.* [49] (C4), and plant photosynthesis follows the Ball–Berry–Woodrow stomatal conductance model [50]. SiBCASA calculates the exchange of water, carbon and energy at a 10 min time step. We use SiBCASA here to specifically assess its tropical drought response, which so far has only been investigated within the SiB component of the model [51,52]. The default SiBCASA model as used in the previous publications cited uses meteorological driver data from the ECMWF ERA-Interim reanalysis [53].

In this work, we improve SiBCASA in two ways: (i) by supplying it with the precipitation product MSWEP which, with PCR-GLOBWB, best reproduces the surface hydrology of the Amazon basin and (ii) by supplying SiBCASA directly with the soil moisture saturation fraction produced by PCR-GLOBWB each day at midnight. PCR-GLOBWB accounts for the run-off and has an improved infiltration scheme. SiBCASA, like several terrestrial biosphere models, simulates the exchange using point simulations, and has a high infiltration capacity and therefore does not account for run-off.

SiBCASA contains 25 soil layers that get progressively larger downwards, with a top layer of 0.02 m and a bottom layer of 3 m, totalling 15 m. To translate soil moisture from PCR-GLOBWB into SiBCASA, we note that PCR-GLOBWB has two distinct layers: 0–0.3 m depth and 0.3–1.5 m depth, which roughly correspond to the first seven SiBCASA soil layers (0–0.28 m) and layers 8–14 (0.28–1.48 m), respectively. Daily PCR-GLOBWB soil moisture saturation fraction fields are spatially averaged to 1×1° resolution to match the resolution in SiBCASA, and these fields are used to adjust SiBCASA's soil moisture in the mentioned layers accordingly.

Electronic supplementary material, §A.7 summarizes the interaction of soil moisture with GPP in SiBCASA. Briefly, soil moisture stress is calculated based on the availability of moisture between wilting point and field capacity, and the presence of roots at each depth of the soil. Limited availability of soil moisture leads to (i) reduced carboxylation capacity *V*_m_, (ii) reduced canopy respiration *R*_d_, (iii) a lowering of the mesophyll conductance (*g*_m_) and (iv) a lowering of the minimal stomatal conductance (*g*_s_, see electronic supplementary material, equation A.6). *V*_m_ subsequently impacts two of the three assimilation rates in the Farquhar *et al.* photosynthesis model: limitations due to Rubisco enzymatic conversion (*ω*_c_, electronic supplementary material, equation A.1) and due to the export capacity for photosynthates (*ω*_s_, electronic supplementary material, equation A.3). The third limiting rate (due to light availability, *ω*_e_, electronic supplementary material, equation A.2) is not affected by soil moisture stress but, like the others, does respond to heat stress that occurs typically during midday. All three rates are further reduced when atmospheric relative humidity decreases (reduced *g*_s_, electronic supplementary material, equation A.6), with the lowest assimilation rate (i.e. most limiting, electronic supplementary material, equation A.5) determining simulated GPP.

In this paper, we compare the results from our default SiBCASA simulations (using ERA-Interim precipitation) with the simulations with MSWEP precipitation, and with the coupled SiBCASA–PCR-GLOBWB system. Additionally, we have created a set of three alternative model realizations with different rooting depths for the plant functional type ‘Evergreen Broadleaf Forest’ (EBF) of 2, 3 and 5 m, recognizing that the soil moisture stress is highly sensitive to this largely unknown parameter. SiBCASA prescribes the rooting density as an exponential function from the surface down to this rooting depth, but any layer with roots present can access soil moisture if available. The values chosen represent the limit of the GPP response, which grows excessively large below 2 m but does not decrease much further above 5 m rooting depth. We use the coupled model with SiBCASA's default rooting depth (3 m) as our main result, and use the range of values with different rooting depths as an uncertainty in the GPP numbers. Further details on model spin-up and set-up are provided in the electronic supplementary material.

### (c) Region definitions

In our analysis, we present our results by aggregated regions within the Legal Amazon (following Gatti *et al.* [18] and Van der Laan-Luijkx *et al.* [24]). The mask file for the Legal Amazon region can be obtained at: https://doi.org/10.18160/P1HW-0PJ6. The subregions are defined based on Köppen–Geiger climate zones [54]. Regions A and B are evergreen forests, with continuously high precipitation or seasonally dry conditions, respectively. Region C has more savannah vegetation, and a strong seasonality in precipitation, and is known as the Brazilian ‘cerrado’.

## 3. Results

In this section, we first analyse the effects of the 2015–2016 El Niño period on precipitation, discharge and soil moisture in the Amazon region using PCR-GLOBWB (§3a), and we subsequently examine the resulting changes in the Amazon carbon balance using the SiBCASA model coupled with PCR-GLOBWB for the soil moisture fields (§3b).

### (a) Impacts on the hydrological balance

The discharge of the South American river systems as calculated by PCR-GLOBWB is shown in figure 1*a*. The main rivers are clearly visible: the Orinoco in the north, the Amazon and its tributaries in the centre, and the Paraña in the south. Feeding into these big rivers are countless smaller streams and rivers. We have used observations from 360 stations to perform an extension of the validation of PCR-GLOBWB presented in Sutanudjaja *et al.* [31] with more recent discharge data and a focus on the Amazon basin. The locations of the stations are included in figure 1*b*, indicating their respective KGE scores (see §2), which confirm that PCR-GLOBWB performs well across the Amazon. Figure 1 also highlights the results at two observation stations: Obidos at the main stem of the Amazon river (figure 1*c*) and Caracarai in the northern part of the basin (figure 1*d*). Obidos is reasonably well reproduced (KGE = 0.53), especially with MSWEP precipitation, although the peaks arrive early and are slightly too high. Caracarai is well simulated, with skill scores well above the average for all basins (KGE = 0.83).

**Figure 2.**
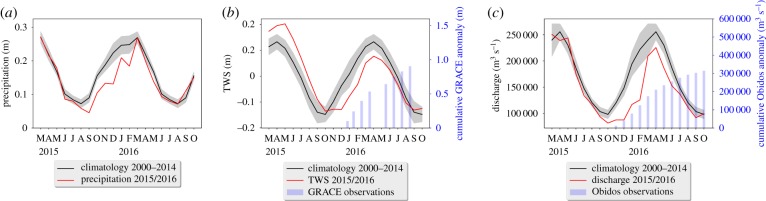
(*a*) Basin-averaged precipitation, (*b*) basin-averaged thickness of total water storage (TWS) anomaly and (*c*) discharge at Obidos from PCR-GLOBWB for the 2015/2016 El Niño period (red). A climatology of previous years 2000–2014 is shown for comparison (black). The PCR-GLOBWB results are from the simulation with MSWEP precipitation. Starting from October 2015, the cumulative anomaly of observed GRACE terrestrial water storage and Obidos discharge observations (where available) are shown in (*b*) and (*c*), respectively.

In September 2015, the monthly precipitation drops to 40–50% below the climatology (average over 2000–2014), maintaining dry season conditions (defined as less than 100 mm precipitation in 30 days) for a month longer than average. Figure 2*a* shows that the Amazon received 220–390 mm (13–22%) less rain between September 2015 and May 2016, leading to reductions of the simulated total water storage (TWS, figure 2*b*). The TWS is significantly higher than the climatology at the start of 2015, but decreases rapidly when the precipitation anomaly starts in September. The TWS remains significantly low until June 2016 and stays below the climatological average until September, a whole year after the start of the anomaly. River discharge is 40% lower than average across the basin between December 2015 and February 2016, and remains 10–20% below average until July 2016, much beyond the persistence of the precipitation anomaly. River discharge at Obidos—the final measurement station of the Amazon river and thus the aggregation of all the run-off in the Amazon basin—was 1100 km^3^ lower than average over the October–April period, which is a reduction of 25% (figure 2*c*). This corresponds to 230 mm less run-off for each square metre of the Obidos catchment, which is comparable to the precipitation reduction described above.

**Figure 3.**
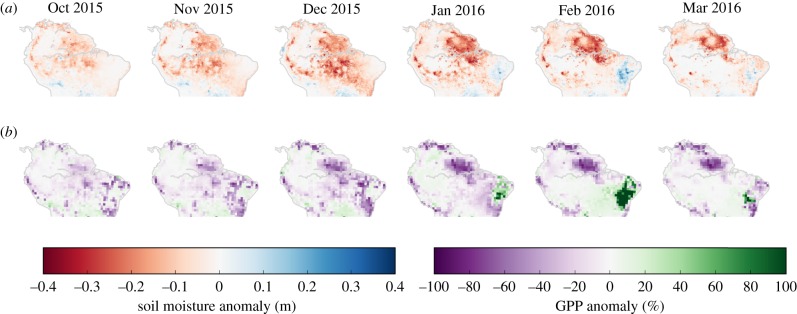
Soil moisture anomalies (in m) per month as calculated by PCR-GLOBWB for the first 1.5 m of the soil profile over the Amazon basin for October 2015–March 2016 in comparison with the climatology over 2000–2014 (*a*), and GPP anomalies (in %) per month in comparison with the climatology over 2009–2014 (*b*).

Figure 3*a* shows the spatial distribution of the anomalies in the monthly soil moisture availability in the first 1.5 m soil depth across the Amazon basin. The soil moisture becomes significantly low in October 2015 and increases to a peak in both area and strength in December 2015/January 2016. During this period, 75% of all simulated gridcells in the Amazon basin have a negative soil moisture anomaly with an average reduction of almost 30%. The largest reductions occur in the eastern part of the basin (>0.3 m less water available than average in the top 1.5 m), but the western part of the basin is also significantly anomalous. The soil moisture storage begins to recover in February 2016, but especially in the north-eastern part the recovery takes longer, and the basin average does not return to the climatological average until September 2016.

### (b) Impact on the gross primary production

The 2015–2016 El Niño reduced GPP across the whole Amazon basin, integrating to a −0.95 PgC of GPP anomaly over the six-month period from October 2015 to March 2016 (table 1). This number has an uncertainty range of nearly 0.5 PgC (−1.20 to −0.69), which is calculated from alternative model realizations with different rooting depths. Most of this anomaly occurred in October to December 2015 (−0.53 (−0.73 to −0.37) PgC), and the January to March anomaly was slightly smaller (−0.42 (−0.47 to −0.32) PgC) in magnitude and uncertainty. Spatially, reductions were widespread across the basin, with the eastern Amazon most strongly impacted (figure 3*b*), correlating significantly (*r* = 0.66, *N* = 2934, *p* < 0.001) with the soil moisture anomalies from figure 3*a*. This suggests a regionally strong impact of soil moisture on GPP, as also seen in the large temporal correlations between their anomalies (electronic supplementary material, figure A.7): 40% of the domain shows correlation coefficients of >0.5 (*N* = 83783), but with a large difference between the different climate zones.

**Table 1.** Changes in GPP (PgC) during the El Niño period in comparison with the baseline years 2007–2014. Values are derived for the whole Amazon basin and by the region. Results from the coupled SiBCASA–PCR-GLOBWB simulations are compared with the default SiBCASA run, which uses ERA-Interim meteorology and no coupling to the PCR-GLOBWB soil moisture. Ranges in parentheses result from different rooting depths in the model, as discussed in the main text.

	SiBCASA-default ([55])	SiBCASA–PCR (this work)		
region	Oct–Mar 2015/2016	Oct–Mar 2015/2016	Oct–Dec 2015	Jan–Mar 2016
Amazon (Legal)	−0.18	−0.95 (−1.20 to −0.69)	−0.53 (−0.73 to −0.37)	−0.42 (−0.47 to −0.32)
Region A (EBF-wet)	+0.04	−0.10 (−0.14 to −0.07)	−0.05 (−0.06 to −0.03)	−0.06 (−0.08 to −0.04)
Region B (EBF-s.dry)	−0.14	−0.52 (−0.66 to −0.37)	−0.28 (−0.39 to −0.19)	−0.24 (−0.28 to −0.18)
Region C (EBF-sav.)	−0.07	−0.30 (−0.37 to −0.23)	−0.19 (−0.26 to −0.14)	−0.11 (−0.11 to −0.09)

**Figure 4.**
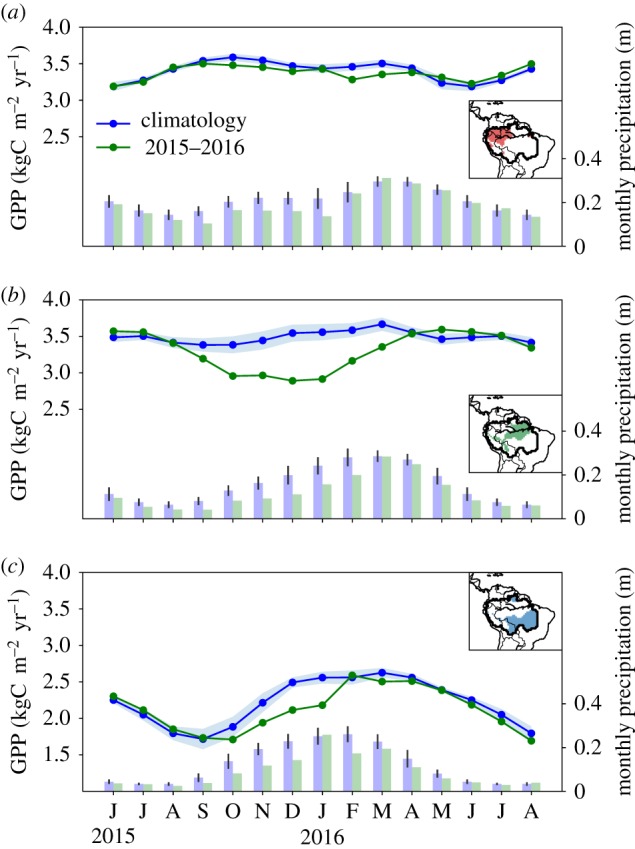
Monthly GPP for the El Niño period June 2015 to August 2016 (green line) for Region A (*a*), Region B (*b*) and Region C (*c*), in comparison with the average GPP climatology over the years 2007–2014 (blue line). Monthly precipitation values are shown for the El Niño period (green bars) and for the climatology over 2000–2014 (blue bars).

Region B contributed most to the GPP reduction (−0.52 (−0.66 to −0.37) PgC), followed by Region C (−0.30 (−0.37 to −0.23) PgC), while contributions from Region A are small (−0.10 (−0.14 to −0.07) PgC) (table 1). Region B is also the first region to show GPP anomalies >1*σ* in September 2015 (figure 4), when precipitation falls below 50% of its climatological amount. Anomalies in precipitation in Region A are similar in absolute amounts by then (40–60 mm), but precipitation rates remain well above 100 mm month^−1^ in this much wetter region. The GPP anomaly of Region A does not exceed 5% of the total during any time in the period August 2015 to February 2016, when precipitation returns to climatological averages.

Not only does the reduction in GPP of Region B start earlier, but also GPP declines more rapidly to a peak anomaly of −19% in December 2015/January 2016. It furthermore remains low until April 2016, one month after the precipitation returns to normal amounts. GPP in Regions A and C goes below 1*σ* in October 2015, and the peak anomaly of Region C (17% below average) occurs in December 2015. GPP in Region C recovers to climatological values in February 2016 following a month of normal rainfall, but returns to slightly below average even in the next dry season period in 2016. In total, the GPP anomaly across the Amazon basin is −0.95 (−1.20 to −0.69) PgC during October 2015 to March 2016 compared with the 2007–2014 average.

**Figure 5.**
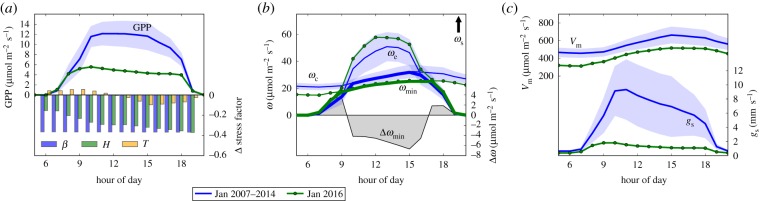
Reduction of key variables over an average diurnal cycle during the month of January 2016 compared with the average of all months of January during 2007–2014, for a selected grid box in Region B. 2016 values are in green and the 2007–2014 average in blue with the shading indicating the 1*σ* s.d. (*a*) GPP and the three stress factors of soil moisture (*β*), humidity (*H*) and heat (*T*) (a reduction in a stress factor equates to an increase in stress); (*b*) *ω*_c_ and *ω*_e_ and their minimum 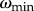, with its reduction during 2016 shown in grey shading (*ω*_s_ is off scale and never limiting in this region, see also electronic supplementary material, equations A1–A5); and (*c*) *V*_m_ and *g*_s_. Note that (*b*) gives the reduction in the assimilation rates (*ω*) in the absence of the humidity stress through *g*_s_ to separate the drought effects on GPP.

So, what drives the reductions in GPP during the 2015/2016 El Niño period? To answer that question, we look at the different components that govern GPP: photosynthetically active radiation (PAR), *V*_m_, humidity stress, soil moisture stress (*β*), heat stress, *g*_m_ and *g*_s_. Their influence on the three limiting assimilation rates (*ω*_e_, *ω*_c_ and *ω*_s_) in the Farquhar *et al.* [48] photosynthesis model used in SiBCASA is briefly summarized in the electronic supplementary material, section A.7, and fully documented in Sellers *et al.* [46] and Suits *et al.* [56]. Figure 5 shows their change from climatological values over an average diurnal cycle in the month of January 2016 for a representatively selected grid box in Region B (the location of which is shown in figure 1*a*). Clearly, the GPP anomaly that we observe in our results is incurred during the daily peak of photosynthesis, with values between 10:00 and 16:00 most strongly reduced by up to 7 μmol m^−2^ s^−1^ (>50%). This is the period of the day that *ω*_c_ limits GPP (figure 5*b*) and thus the *enhancement* of light-limited GPP (*ω*_e_) due to the availability of extra PAR during January 2016 does not lead to increased GPP. Instead, we find that the GPP reduction from climatology is strongly controlled by (i) humidity stress, which reduces stomatal conductance (figure 5*c*), and (ii) soil moisture stress and heat stress, which reduce the maximum carboxylation rate *V*_max_ and mesophyll conductance (not shown). The reduction in 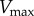 causes a reduction of the Rubisco-limited assimilation rate (*ω*_c_) by up to 6 μmol m^−2^ s^−1^, which, when multiplied with the fraction of absorbed PAR (approx. 0.5), accounts for as much as 40% of the total daily GPP reduction during this month. The total reduction in GPP combines effects (i) and (ii), balancing lowered assimilation rates with lowered *g*_s_ and *g*_m_.

By March 2016, the picture shown in figure 5 has changed (see electronic supplementary material, figure A.8). Although the carboxylation rate and *g*_m_ are still reduced owing to continuing anomalous soil moisture stress at this location, the heat and relative humidity stress follow the return to near-normal conditions in the atmosphere, as also indicated by the potential evapotranspiration (electronic supplementary material, figure A.11). In this regime, diurnally declining *g*_s_ and increasing heat stress still play an important role in shaping GPP, but their values are now much closer to the climatological 1*σ* variability. What remains is a small GPP anomaly (2 μmol m^−2^ s^−1^), attributable to the reduced *g*_m_ and *ω*_c_ as Rubisco-enzyme activity still suffers from low soil moisture levels. All anomalies have disappeared by April 2016 (see electronic supplementary material, figure A.9).

## 4. Discussion

An important source of uncertainty in simulating tropical surface hydrology and carbon cycling is the availability of water from precipitation. Various precipitation datasets are available, each with its own strengths and weaknesses in tropical regions. In a comparison by Sun *et al.* [57], datasets that included surface gauge observations tended to perform better than satellite-based datasets, while meteorological reanalyses ‘show great inconsistency in their annual precipitation amounts’. In this work, we used one of each type of dataset (ERA5, TRMM, MSWEP) and first assessed them against discharge data specifically in our region of interest, providing a solid base for our investigation of carbon cycle impacts. Our final choice of precipitation driver dataset for PCR-GLOBWB (MSWEP) falls in the middle of the range for annual precipitation, but is still substantially lower than ERA-Interim, which suffers from too high rainfall in South America, like most reanalysis products [57].

Replacing the ERA-Interim precipitation with MSWEP values already triggers more drought stress and GPP reductions in SiBCASA, even before replacing its soil moisture with that from PCR-GLOBWB. We find GPP reductions of 0.73 PgC for the Amazon when using the soil moisture from SiBCASA that results from MSWEP precipitation (electronic supplementary material, table A.1). This number is the same as when using ERA-Interim precipitation and PCR-GLOBWB soil moisture (0.73 PgC), suggesting that the largest effect indeed is from the lower precipitation amount of MSWEP. The combined use of PCR-GLOBWB soil moisture and MSWEP precipitation makes the anomaly even larger (0.95 PgC), because precipitation also affects the relative humidity of SiBCASA's canopy. PCR-GLOBWB soil moisture also has different spatial patterns compared with the soil moisture resulting from SiBCASA and MSWEP precipitation. We furthermore note that the latter combination cannot be validated with discharge observations, and it is only through the use of PCR-GLOBWB that we could increase our faith in the MSWEP product as a driver for this study.

We report a −0.95 (−1.20 to −0.69) PgC anomaly in GPP over the period October 2015 to March 2016, due to the drought conditions during the El Niño period. Outside this window, anomalies are small, and integrating over different time periods such as the year 2015 (−0.72 (−0.99 to −0.50) PgC), or the period September 2015 to June 2016 (−0.97 (−1.25 to −0.70) PgC), does not change this anomaly much. Our number is therefore in a similar range compared with other studies. Liu *et al.* [16] report a 0.9 PgC reduction of GPP over tropical South America in 2015, relative to the year 2011 and integrated over a nearly 40% larger area than we report on here. Gloor *et al.* [58] report a 0.9 PgC reduction of GPP based on GoSAT SIF data and over the three-month period October to December 2015 only, but their integration area is also larger and includes some strong GPP anomalies just outside the Legal Amazon mask. Our estimate of GPP reduction during the peak of the El Niño period (October–December 2015) of 0.53 (0.37 to 0.73) PgC also agrees reasonably well with an independently derived estimate using SIF. Koren *et al.* [27] report a reduction of 0.34 to 0.48 PgC for the same period and region, and the spatiotemporal patterns also correspond well even though our calculations are completely independent of this space-based view of GPP.

We find that the reduction of GPP is accompanied by a reduction in respiration (*R*) in SiBCASA, such that net ecosystem production (NEP = GPP − *R*_autotrophic_ − *R*_heterotrophic_) gives an additional release of carbon across the Amazon of 0.32 (0.20 to 0.45) PgC over the six months considered here. This is in line with atmospheric CO_2_-based estimates of Gloor *et al.* [58] (0.5 PgC over 10 months), but different from those of Liu *et al.* [16], who found a total 0.9 PgC anomaly in net biome exchange (NEP+fire losses), equal to their total GPP anomaly as CO_2_ release from fires and respiration changed only little in their analysis. We note though that translation of a GPP anomaly to NEP involves assumptions on the partitioning of GPP into net primary production (NPP) and of NPP into the different carbon pools that respire at different rates. If we allowed NPP allocation patterns to change during droughts as was found by Doughty *et al.* [59], or if we would allow carbon-use efficiency (NPP/(NPP + *R*_a_)) to decrease as was found by Metcalfe *et al.* [60], it could substantially change the NEP anomaly calculated with SiBCASA.

Our results confirm that rooting depth is a highly uncertain but influential parameter in the drought response of GPP [61–65]. We used rooting depths between 2 and 5 m and this resulted in a nearly 0.5 PgC range in the GPP anomaly, while further increases of the rooting depth have little further effect (not shown). The rooting depth in SiBCASA is prescribed per plant functional type (PFT) and does not change over time, while PFTs are based on the dominant vegetation type per 1 × 1° gridbox, which is ‘Evergreen Broadleaf Forest’ over much of the domain (66%) considered. SiBCASA thus contains very little spatial differentiation in its rooting depth, and vegetation is assumed to have access to all available water across this depth. By contrast, PCR-GLOBWB includes up to six different vegetation classes per 1 km^2^ to prescribe the rooting depth and also includes the root density profile to determine how much soil water can be accessed for transpiration [66]. A recently released rooting depth estimate [67], as well as earlier studies [51,52], suggests that especially the seasonally dry rainforest that falls within our Region B generally has deeper roots (>10 m) than the rainforest in our Region A—much deeper than prescribed in this work. This part of the forest would therefore be less affected by changes in soil moisture in the upper layers, and including such regional details in SiBCASA could decrease our estimated GPP anomaly for Region B. Improving our knowledge on the *access* to soil moisture by vegetation might therefore constitute a next challenge for the Amazon region.

## 5. Conclusion

We show that GPP in the Amazon reduced by 0.95 (0.69 to 1.20) PgC during the 2015/2016 El Niño period compared with the 2007–2014 average, with the reduction during October–December 2015 totalling 0.53 (0.37 to 0.73) PgC. There were significant differences between subregions: the north-western region is least affected by the drought (11% (10–12%) of the Amazon total anomaly), whereas the eastern and southern regions experience strong reductions in GPP (56% (54–56%) and 33% (31–33%), respectively). In the southern region, which has the most pronounced dry season and more savannah vegetation, the reduction is caused by a combination of higher than normal vapour pressure deficit and soil moisture stress. The latter contributes even more to the GPP decrease in the eastern seasonally dry tropical forest region, where we illustrated the mechanism of GPP reduction in great detail. We note that the influence of assumed rooting depth on the calculated anomalies is large and could especially have affected our simulations for Region B.

Confidence in our simulated soil moisture comes from the hydrological model PCR-GLOBWB in combination with MSWEP precipitation, which simulates the discharge in the Amazon region well in comparison with observations at many stations across the basin. Soil moisture stress during the El Niño period extended across the entire region and persisted especially long in the north-eastern part of the Amazon. Implementing this soil moisture stress in SiBCASA to replace the default parameterization, and/or using the MSWEP precipitation dataset that we validated with PCR-GLOBWB, significantly enhances the estimated reductions in GPP.

## Supplementary Material

Supplementary material

## Data accessibility

The model PCR-GLOBWB is freely available at https://github.com/UU-Hydro/PCR-GLOBWB_model. Model results are available at https://doi.org/10.18160/6J39-P8YE.
