# Supplementary material for: Changes in surface hydrology, soil moisture and gross primary production in the Amazon during the 2015/2016 El Niño
Source: Philos Trans R Soc Lond B Biol Sci. 2018 Oct 8;373(1760):20180084. doi: 10.1098/rstb.2018.0084 (PMC6178443; doi:10.1098/rstb.2018.0084)
Supplement: Supplementary material [file rstb20180084supp1.pdf]

Supplementary Materials for: "Changes in surface hydrology, soil moisture, and Gross Primary Productivity in the Amazon during the 2015/2016 El Niño "

Erik van Schaik[a], Lars Killaars[b], Naomi E. Smith[a], Gerbrand Koren[a], L.P.H. (Rens) van Beek[c], Wouter Peters[a,b], Ingrid T. van der Laan-Luijkx[a]

[a] *Wageningen University and Research, Wageningen, The Netherlands*

[b] *University of Groningen, Centre for Isotope Research, Groningen, The Netherlands*

[c] *Utrecht University, Department of Physical Geography, Faculty of Geosciences, Utrecht, The Netherlands*

Email address: erik.vanschaik@wur.nl (Erik van Schaik)

Appendix A. Supplementary Materials

Appendix A.1. Validation method of PCR-GLOBWB

In this supplement, we use the monthly results of PCR-GLOBWB together with discharge measurements from the Global Runoff Data Centre [1] and the HYBAM dataset (`https://www.ore-hybam.org`) to validate PCR-GLOBWB. Accurate observations to validate hydrological models for parameters such as evapotranspiration and soil moisture are scarce, especially for the tropics. It is therefore difficult to accurately validate a hydrological model with observations of these parameters. A possible solution is to compare results to discharge observations, as these observations are an aggregation of the total water balance of the catchment. An advantage of PCR-GLOBWB is that it contains a routing module whereby runoff generated by the grid cells is routed along a river network and can be compared to discharge observations. In northern South America, several discharge observation stations are present that can be used for the validation of PCR-GLOBWB. In total, more than 350 stations are used, with the majority provided by the GRDC dataset. Not all stations are available for the entire period (2000-2016). There are 350 stations present that have data available in 2000. This decreases to 303 in 2007, 177 in 2010 and only 22 have data from 2011 up to 2016 (Figure A.2 shows this decline). 12 stations are chosen from the HYBAM dataset, all with continuous monthly data available for the years 2000-2016 (or 2003-2016 for the Ciudad Bolivar station). The locations of the stations in South America are shown in Figure A.1. Each station needs to have at least 12 months of discharge data to be included in the analysis. This reduces the amount of stations available for the validation of the ERA5 run, but also allows for more meaningful statistics, as it is not correct to give a station with only 1 measurement the same weight as a station with a continuous record.

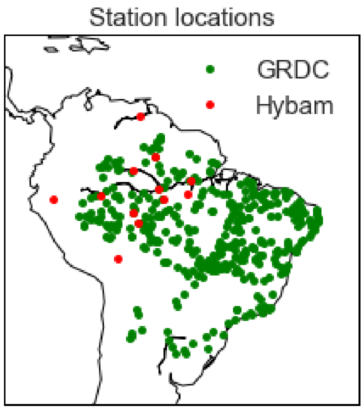

Figure A.1: Location of stations used in the validation of PCR-GLOBWB.

To evaluate the skill of PCR-GLOBWB, Kling-Gupta Efficiency (KGE, [2]), correlation and anomaly correlation scores are calculated using the simulated and observed discharge for each of the stations. KGE equally measures bias, timing differences and amplitude differences. The score varies between 1 and minus infinity, where 1 denotes a perfect match of the model with the observations and negative scores are considered a poor comparison. In addition, the

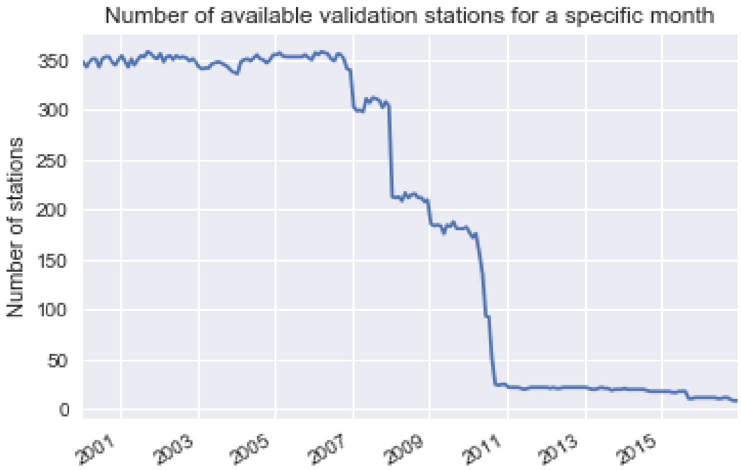

Figure A.2: Number of available stations for a specific month for the period 2000-2016.

correlation and anomaly correlation are used to evaluate the skill of the model regarding the timing of both the seasonal cycle and the timing of seasonal and interannual anomalies, such as the El Niño event.

Appendix A.2. Model performance PCR-GLOBWB

The histograms of Figure A.3-A.5 show the distribution of the skill scores for all the used stations in South America. Most of the stations have a positive KGE score, with the majority of the scores being around 0.5. There are some stations with a negative score, but they are mostly located in the coastal region or at the western edges of the Amazon catchment (Figure 1B in the main text). PCR-GLOBWB generally has a high correlation with the observations, with 50% of the studied basins scoring above 0.78 and 25% scoring above 0.86. Since the correlation is also included in the KGE score, a correlation higher than the KGE score indicates that the bias and amplitude difference are worse than the correlation. These two indexes compare the quantity of water to the observations and are mostly governed by (meteorological) input information. The anomaly correlation is lower than the normal correlation, indicating that the seasonal cycle determines part of the skill of the model. However, the anomaly correlation remains high in most of the basins: 50% of the basins have an anomaly correlation higher than 0.6 and 25% have values higher than 0.75. The distribution of skill scores is similar for the different precipitation simulations, the 25%, median and 75% percentile of the ERA5, MSWEP and TRMM simulations are within a few percent of each other. The MSWEP simulation scores slightly higher when all available stations are used and is therefore selected for the SiBCASA simulations.

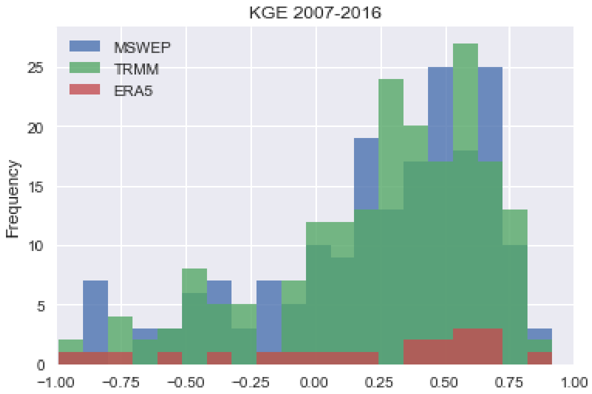

Figure A.3: Histogram of the KGE scores of all used stations that have >12 months of data in the period 2007-2016.

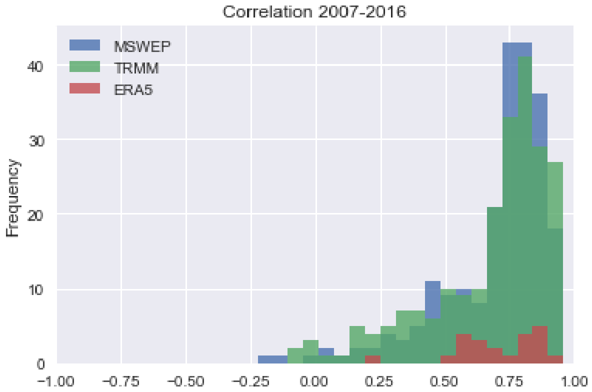

Figure A.4: Histogram of the correlation scores of all used stations that have >12 months of data in the period 2007-2016.

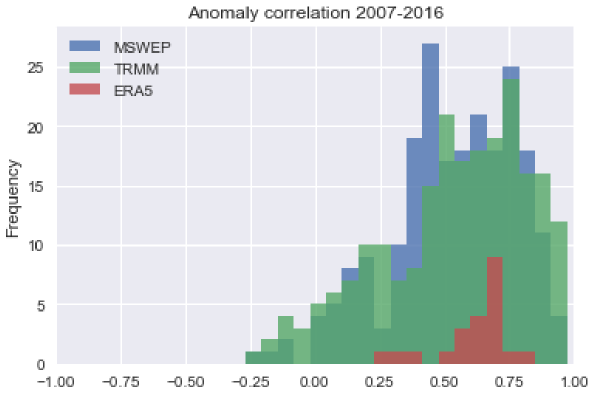

Figure A.5: Histogram of the anomaly correlation scores of all used stations that have >12 months of data in the period 2007-2016.

Appendix A.3. Spin-up and set-up of PCR-GLOBWB

The Amazon basin has a long groundwater response time due to the large size of the basin. The model was therefore spun-up once from 1970 to 2010 followed by another run from 1970 to 2000, which totals to a 70 year spin-up. After the spin-up, the model was run three times: once for each precipitation dataset. These runs had alternative start years of 2000, 2007 and 2010 for MSWEP, TRMM and ERA5 respectively due to the availability of the input data sets, and continued until the end of 2016. MSWEP, with its longest record, provides the initial conditions for the other 2 runs.

Appendix A.4. Spin-up and set-up of SiBCASA

The model spin-up has been done first for the period 1850-1870 to reach an equilibrium in the carbon pools and secondly during 1870-1999 using historical CO_2 data as in van der Velde et al. [3]. During this spin up we use recycled meteorological input data from the recent years. All meteorological input data comes from ERA Interim [4] during spin-up, while during our final simulations which cover 2000-2016 we have used either ERA-Interim or MSWEP precipitation. Following the available archive, we have used hourly MSWEP precipitation for 2013-2016 and hourly values scaled from MSWEP daily values for 2000-2013. We have run SiBCASA on a one-degree resolution for the South American domain and saved monthly output. We calculated all anomalies over the 2015/2016 El Niño period in comparison to the climatological average over 2007-2014.

Appendix A.5. Discharge sensitivity tests

Figure 1C in the main text shows the discharge at Obidos in Brazil. PCR-GLOBWB systematically simulates the peak flow earlier than observed. This is also found in a lower resolution of PCR-GLOBWB (0.5 degree grid boxes) and [5] tried to improve these discharge results by coupling the hydrological model to the hydrodynamic routing scheme Delft 3D [6]. They hypothesized that this mismatch was due to their coarse resolution and because of its kinematic wave approximation of surface water flow in an area with limited topographic gradients. Our model has a higher resolution (1/12th of a degree), but the same problem occurs here. PCR-GLOBWB coupled to Delft 3D results in a better match to observations, mainly due to increased skill in simulating the inundation of the floodplain, the smaller channels that are present near the main river and the corresponding delay of the discharge peak. We tried to do a simple test to find the sensitivity of the discharge peak to the roughness of the basin and did several simulations with MSWEP precipitation. The results are shown in Figure A.6. It is clear that increasing the surface roughness of PCR-GLOBWB delays the discharge peak and reduces both extremes, the water is more evenly distributed. The simulation with a surface roughness coefficient of 0.45 has both the highest correlation as KGE score to the observations (0.82, 0.78 respectively), but, while the peaks match, the amount of discharge during high and low flood compares less well to the observations. We also want to note that improving the discharge scores by changing the roughness coefficient does not change the amount of soil moisture, as there is no feedback from the inundated floodplains to the soil moisture.

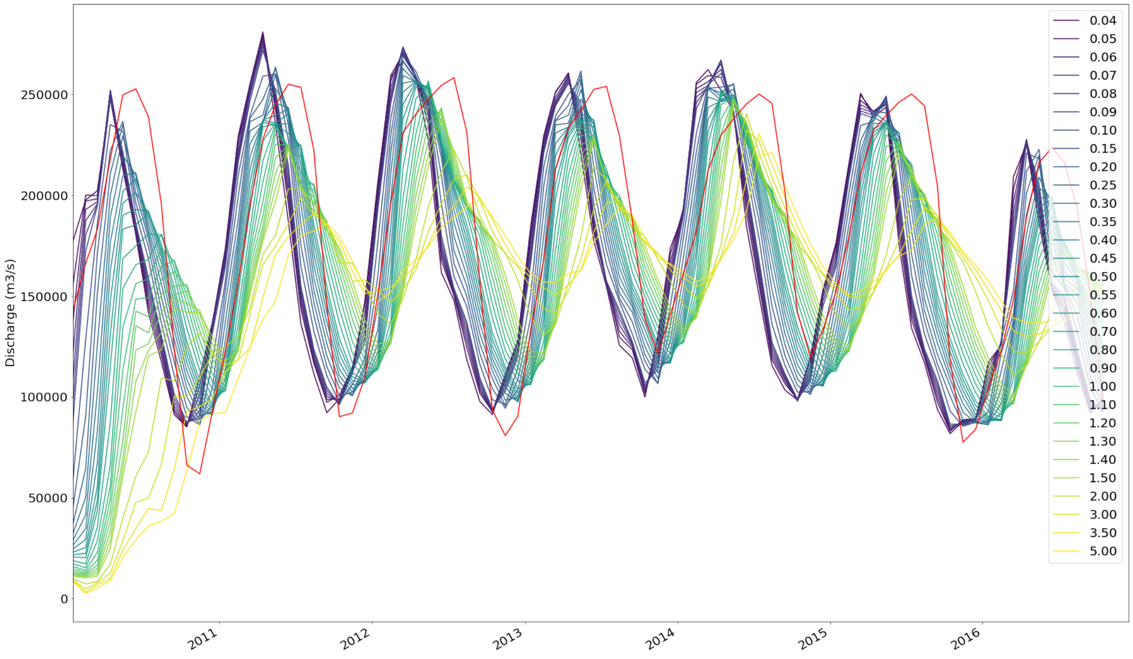

Figure A.6: Discharge at Obidos for simulations with different Mannings roughness coefficients

Appendix A.6. Gross primary productivity (GPP) anomalies

This section includes the correlations between GPP and soil moisture (Figure A.7), and results from additional simulations with SiBCASA (Table A.1. These additional SiBCASA simulations are those coupled to PCR-GLOBWB but driven with ERA-Interim precipitation driver data instead of MSWEP driver data, as well as the SiBCASA simulations with MSWEP precipitation, but without coupling to PCR-GLOBWB.)

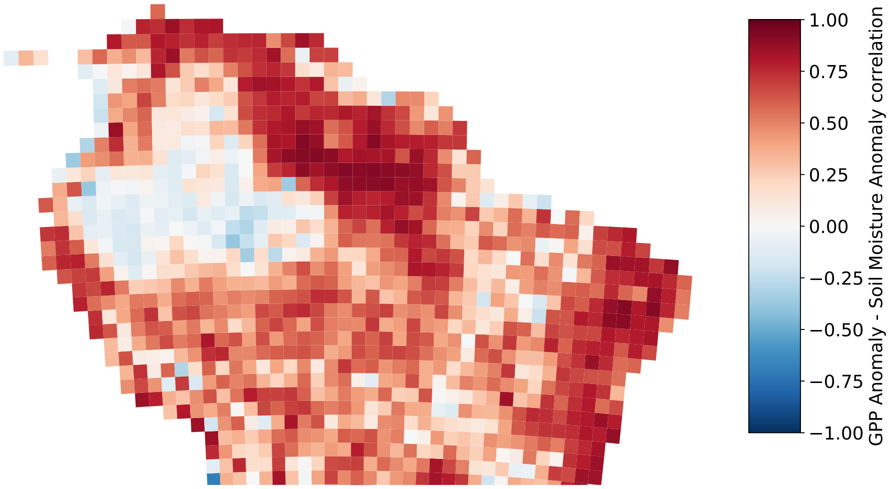

Figure A.7: Spatial distribution of temporal correlations of the GPP anomaly in SiBCASA and the Soil Moisture anomaly in PCR-GLOBWB of the period 2007-2014.

GPP anom. (PgC)	SiBCASA (No PCR). MSWEP precip.	SiBCASA-PCR MSWEP (PCR) and ERA (SiBCASA) precip.		
	Oct-Mar 15/16	Oct-Mar 15/16	Jan-Mar 2016	Oct-Dec 2015
Amazon (Legal)	-0.73	-0.73 (-0.99 – -0.48)	-0.31 (-0.37 – -0.21)	-0.42 (-0.62 – -0.27)
Region A (EBF-wet)	-0.08	-0.04 (-0.07 – -0.01)	-0.02 (-0.04 – -0.00)	-0.02 (-0.03 – -0.00)
Region B (EBF-s.dry)	-0.44	-0.42 (-0.58 – -0.28)	-0.19 (-0.23 – -0.14)	-0.23 (-0.34 – -0.15)
Region C (EBF-Sav.)	-0.20	-0.26 (-0.32 – -0.18)	-0.09 (-0.09 – -0.07)	-0.17 (-0.23 – -0.11)

Table A.1: Reductions in gross primary productivity (GPP) during the El Niño period in comparison to the baseline years 2007-2014. These results are from the SiBCASA simulations with MSWEP precipitation without the coupling to PCR-GLOBWB (second column) and from the SiBCASA runs with soil moisture field from PCR-GLOBWB (driven by MSWEP precipitation) with ERA-Interim precipitation fields used in SiBCASA (columns 3-5). Values are given for the complete period (October 2015 - March 2016) as well as OND 2015 and JFM 2016 separately and are derived for the whole Amazon basin and by region.

Appendix A.7. Photosynthesis model

The photosynthesis model of SiBCASA follows closely the work of Sellers et al. [7], Collatz et al. [8], Farquhar et al. [9], and is briefly summarized here for reference. The principle behind the scheme is that one of three photosynthetic rates is the limiting factor on assimilation. The three assimilation rates in the Farquhar et al. photosynthesis model describe limitations due to Rubisco enzymatic conversion (ω_c), due to the export capacity for photosynthates (ω_s), and due to light availability (ω_e). As per Equations 18-20 of [7], these three limiting rates (mol m^2 s^{-1}) are calculated using

$$\omega_c = f_c(V_m, c_i, O_2) \tag{A.1}$$
$$\omega_e = f_e(\epsilon \mathbf{F} \cdot \mathbf{n}, c_i) \tag{A.2}$$
$$\omega_s = f_s(V_m), \tag{A.3}$$

where c_i is the partial pressure of CO_2 within the leaf (Pa), O_2 is the partial pressure of O_2 within the leaf (Pa), ϵ is the quantum efficiency for CO_2 uptake (mol mol^{-1} or mol J^{-1}), $\mathbf{F}$ is the incident flux of PAR on the leaf (W m^{-2}), $\mathbf{n}$ is the vector normal to the leaf's surface. The functions $f_{c,e,s}$ are described in full in Sellers et al. [7]. The maximum catalytic capacity of Rubsico, V_m (mol m^2 s^{-1}), is given by

$$V_m = V_{\max} f_T(T_c) f_w(W_i), \tag{A.4}$$

where T_c is the temperature at the top of the canopy (K) and W_i is the soil wetness integrated over the root zone. This corresponds to Equation C17 of Sellers et al. [7].

The assimilation rate A (mol m^2 s^{-1}) is then bounded above by the minimum of these, such that

$$A \leq \min(\omega_c, \omega_e, \omega_s). \tag{A.5}$$

To find A, a smoothing is applied to the minimum of the three limiting rates following Equations C6a and C6b of [7].

Stomatal conductance (mol m^2 s^{-1} or m s^{-1}) is largely dependent on the relative humidity around the leaf, and is given by

$$g_s = m \frac{A_n}{c_s} h_s p + b f_w(W_i), \tag{A.6}$$

or Equation C9 of Sellers et al. [7], where m and b are empirically-derived coefficients dependent on vegetation type, A_n is the net assimilation rate (mol m^2 s^{-1}), c_s is the partial pressure of CO_2 at the leaf surface (Pa), h_s is the relative humidity at the leaf surface, and p is the atmospheric pressure (Pa).

The leaf-level assimilation rate from A.5 is converted into a net assimilation rate at the top of the canopy by subtracting canopy respiration, and subsequently integrated to a canopy total (see Sellers et al., 1996, Eq C12-C16). This is the GPP used from the SiBCASA model throughout the main text and Figure 4 and 5A in the main text. Panel B and C of Figure 5 in the main text shows the result of the equations A1-A6 above.

Note that the soil wetness W_2 used in Eq A4 and A6 is in this work derived from either SiBCASA itself, or replaced by the results from the PCR-GLOBWB model.

The mesophyll conductance also depends on soil moisture, via

$$g_m = 4000 V_{\mathrm{max}_0} \Pi f_w(W_i), \tag{A.7}$$

where the coefficient 4000 accounts for the associated drop in CO_2 pressure at high rates of photosynthesis, the subscript 0 denotes the value at the top of the canopy, Π is represents the photosynthetic rate integrated across the canopy, and $f_w(W_i)$ is the soil moisture stress.

Appendix A.8. Key variables explaining the GPP anomalies

This section includes more details on the analysis shown in the main text in Figure 5, which focused on January. Here, we show the same figure for March (Figure A.8) and April (Figure A.9). The monthly evolution of the β and humidity stress factors by region are shown in Figure A.10.

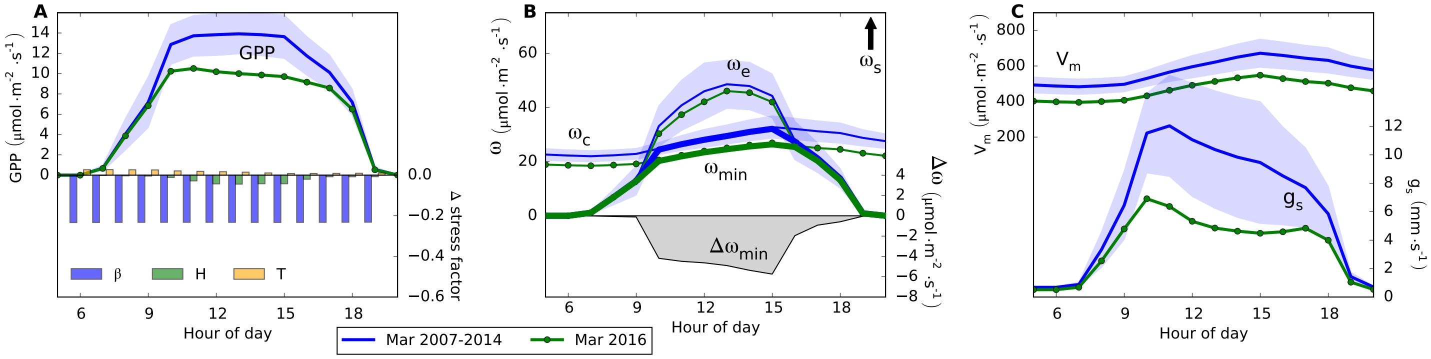

Figure A.8: Reduction of key variables over an average diurnal cycle during the month of March 2016 compared to the average of all months of March during 2007-2014, for a selected grid box in Region B. (Panel A) GPP (2016 in green and the 2007-2014 average in blue with the shading indicating the 1σ standard deviation) and the reductions in the three stress factors for soil moisture (β), humidity (H), and heat (T) (a reduction in a stress factor equates to an increase in stress); (Panel B) ω_c and ω_e and their minimum ω_{min}, with its reductions during 2016 shown in grey shading (ω_s is off scale and never limiting in this region; see also Eq. A1-A5), and (Panel C) V$_m$ and g$_s$. Note that panel B gives the reduction in ω's in absence of the humidity stress through g$_s$ to separately show the drought effects on GPP.

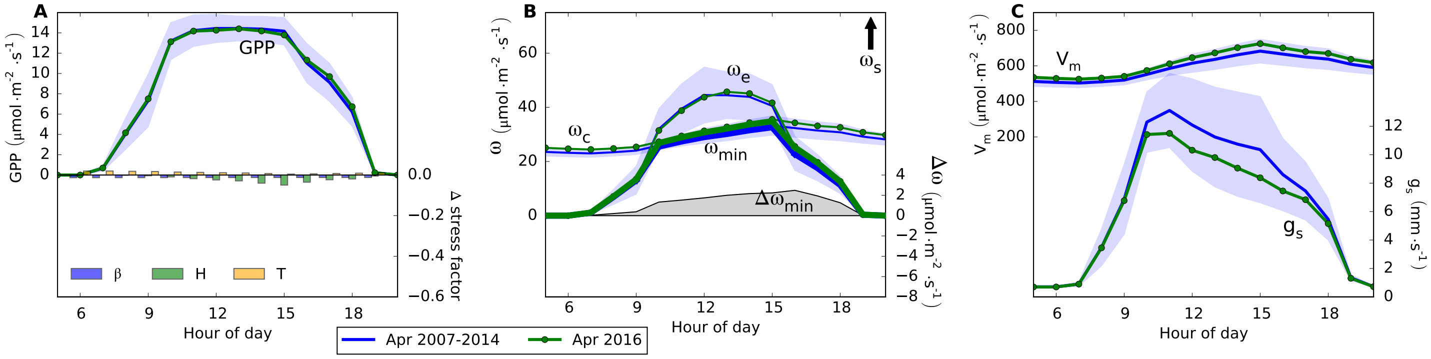

Figure A.9: The same as for Figure A.9, but for April 2016 instead of March 2016.

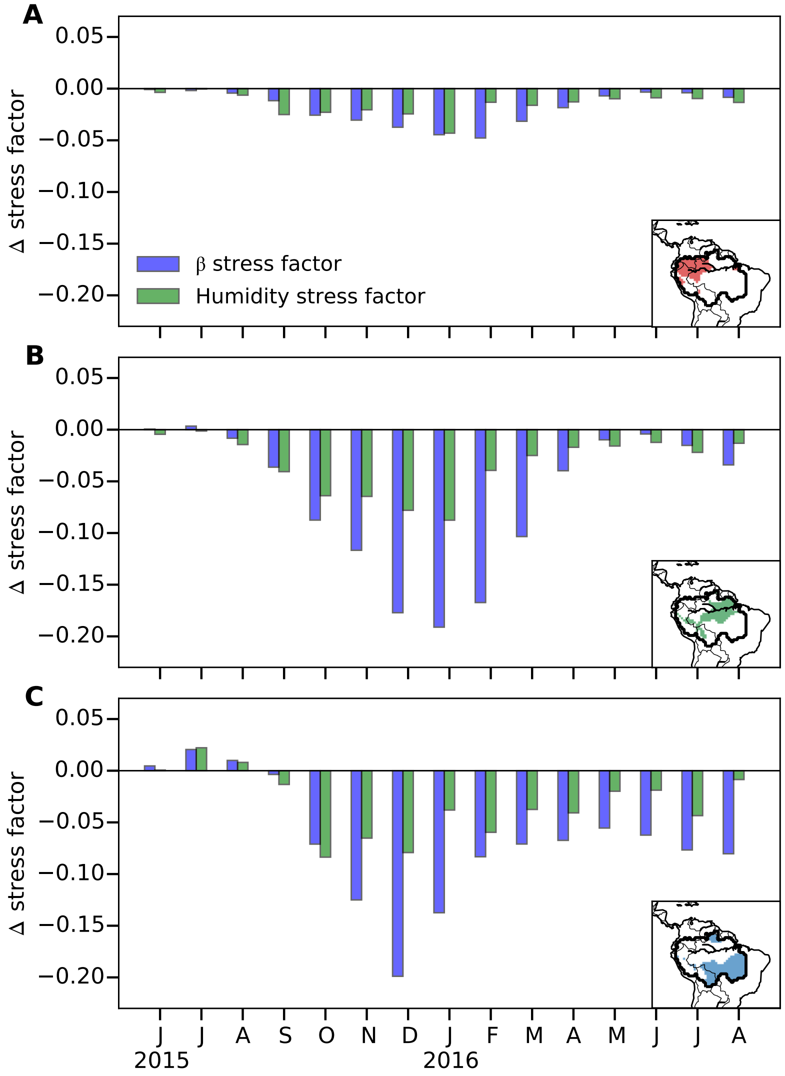

Figure A.10: The change in the monthly stress factors for β (blue) and humidity (green) for the El Niño period June 2015 to August 2016 for Region A (panel A), Region B (panel B), and Region C (panel C), in comparison to the average climatology over the years 2007-2014. Note that the stress factors are averaged over the monthly periods, and therefore also include the night time values which do not impact GPP. For this reason, the temperature stress factor is not shown, as this stress factor has a large diurnal cycle. For β this does not matter, as the soil moisture is given a daily value from PCR-GLOBWB, and the effect on the humidity stress factor is small.

Appendix A.9. Potential evapotranspiration

Figure A.11 shows the potential evapotranspiration as calculated by the Penman Monteith equation [10] using ERA5 data.

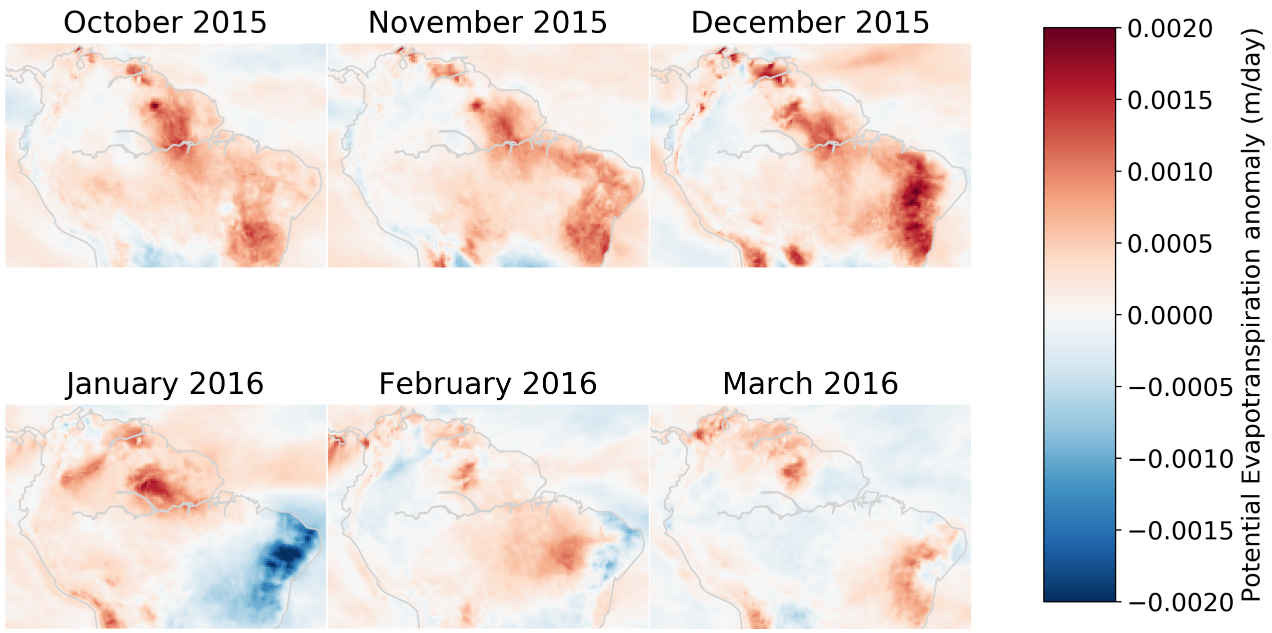

Figure A.11: Potential evapotranspiration anomaly during the El Niño period in comparison to the climatology of 2010-2014.

References

[1] GRDC. The global runoff database and river discharge data. available at: `http://www.bafg.de/GRDC`. obtained: 2 march 2018. 2018.

[2] Harald Kling, Martin Fuchs, and Maria Paulin. Runoff conditions in the upper danube basin under an ensemble of climate change scenarios. *Journal of Hydrology*, 424:264–277, 2012.

[3] IR Van der Velde, JB Miller, K Schaefer, KA Masarie, S Denning, JWC White, PP Tans, MC Krol, and W Peters. Biosphere model simulations of interannual variability in terrestrial 13c/12c exchange. *Global Biogeochemical Cycles*, 27(3):637–649, 2013.

[4] Dick P Dee, S M Uppala, AJ Simmons, Paul Berrisford, P Poli, S Kobayashi, U Andrae, MA Balmaseda, G Balsamo, d P Bauer, et al. The era-interim reanalysis: Configuration and performance of the data assimilation system. *Quarterly Journal of the royal meteorological society*, 137(656):553–597, 2011.

[5] Jannis M Hoch, Arjen V Haag, Arthur van Dam, Hessel C Winsemius, Ludovicus PH van Beek, and Marc FP Bierkens. Assessing the impact of hydrodynamics on large-scale flood wave propagation–a case study for the amazon basin. *Hydrology and Earth System Sciences*, 21(1):117–132, 2017.

[6] Herman WJ Kernkamp, Arthur Van Dam, Guus S Stelling, and Erik D de Goede. Efficient scheme for the shallow water equations on unstructured grids with application to the continental shelf. *Ocean Dynamics*, 61(8):1175–1188, 2011.

[7] PJ Sellers, DA Randall, GJ Collatz, JA Berry, CB Field, DA Dazlich, C Zhang, GD Collelo, and L Bounoua. A revised land surface parameterization (sib2) for atmospheric gcms. part i: Model formulation. *Journal of climate*, 9(4):676–705, 1996.

[8] Go J Collatz, M Ribas-Carbo, and JA Berry. Coupled photosynthesis-stomatal conductance model for leaves of c4 plants. *Functional Plant Biology*, 19(5):519–538, 1992.

[9] GD v Farquhar, S von von Caemmerer, and JA Berry. A biochemical model of photosynthetic co2 assimilation in leaves of c3 species. *Planta*, 149(1):78–90, 1980.

[10] RG Allen, LS Pereira, D Raes, and M Smith. Guidelines for computing crop water requirements-fao irrigation and drainage paper 56, fao-food and agriculture organisation of the united nations, rome (http://www. fao. org/docrep) arpav (2000), la caratterizzazione climatica della regione veneto, quaderni per. *Geophysics*, 156:178, 1998.
